# American Medical Association

**Published:** 1872-06

**Authors:** 


ATLANTA

### Medical and Surgical Journal.

Vol. X.—JUNE, 1872.—No. 3.

---

### ORIGINAL COMMUNICATIONS.

---

### AMERICAN MEDICAL ASSOCIATION.

*MINUTES OF THE TWENTY-THIRD ANNUAL MEETING, HELD IN THE CITY OF PHILADELPHIA, MAY* 7, 8, 9 *and* 10, 1872.

The Association assembled at Horticultural Hall, on Tuesday, May 7, at 11 A.M., and was called to order by the President, David W. Yandell, M.D., of Kentucky—assisted by Vice-Presidents Thomas M. Logan, M.D., of California; Chas. M. Ives, M.D., of Connecticut; R. F. Michel, of Alabama, and J. K. Bartlett, M.D., of Wisconsin.

The Permanent Secretary, W. B. Atkinson, M.D., the Assistant Secretary, D. Murray Cheston, M.D., and the Treasurer, Casper Wister, M.D., all of Pennsylvania, and the Librarian, F. A. Ashford, M.D., of the District of Columbia, were also present.

The proceedings were opened with prayer by the Right Rev. Wm. Bacon Stevens, M.D., D.D., LL.D., Bishop of the Protestant Episcopal Church in Pennsylvania.

Prof. Robert E. Rogers, M.D., on behalf of the profession of Philadelphia, welcomed the delegates in the following remarks:

*Gentlemen Delegates to the American Medical Association,*—It is with unfeigned pleasure that, as Chairman of the Committee of Reception, and on behalf of the medical profession of Philadelphia, I extend to you a sincere and cordial welcome.

Gathered to-day in this hall are representatives of our profession from every quarter of this vast country, reaching from the city of the "Golden Gate" to the Capes of the Chesapeake, from the granite hills of Maine to the waters of the Gulf of Mexico, covering a domain of twenty-four parallels of latitude and fifty degrees of longitude, and embracing a population, of whose health its members are the guardians, numbering not less than thirty-eight millions of men, women and children.

With these boundaries, this area possesses features of climate, a meteorology and a geology more diversified and extensive than those of any other single country on the face of the earth. What may we not hope of benefit to science and humanity by observations made upon these in their connection with disease and hygiene by so many intelligent laborers in the field?

Meeting here on this twenty-fifth anniversary of the organization of the American Medical Association, there are brought together men of every type, of high tone, loyal to their profession, strong men and true—men of sparkling genius, of varied and solid attainments—those who have grown gray in their vocation, laden with the golden harvest of a life's long experience, and those blessed with youth, energy and ambition, engaged in laborious research, all ready to lay their individual contributions upon the altar of our profession.

In view of the reflections which here arise, what a spectacle of "moral grandeur" in possible achievements in the cause of suffering humanity does this assemblage present.

More than any other department of human knowledge, does medicine appropriate the laws of the other positive sciences as parts of its organic whole, modifying and assimilating them, finding in them sustenance and warmth and life. It is, indeed, the highest application of the truths of the other departments of knowledge directed to the beneficent end of preserving health and assuaging physical pain. It may, perhaps, be not unaptly compared to some *vast sea*, receiving the contributions from a thousand perennial streams flowing from distant and widely-separated regions, and then dispersing the commingled waters on the wings of the ever-moving winds, to spread their vivifying, renovating showers over the wide circuit of the globe.

Since the organization of our Association, as by curious coincidence, a new era has been inaugurated; marvelous progress has been made in the development of science, bearing upon human comfort and the mitigation of disease. Thus the electric telegraph, the vast extension of the iron track, the introduction of anæsthesia, and the device of various instruments of precision for the exploration and cure of the maladies of our race. These grand opportunities and great advances have been within the reach of all; therefore, as a natural result, our numbers have continued to increase, and the value of our transactions greatly to improve, thus securing an unabated prosperity to our organization.

Whatever qualifications may be made as to the amount of positive knowledge contributed through such annually-recurring gatherings as these, it can not be denied that they exercise, personally, a most wholesome and elevating influence upon the members of the profession. They serve to strengthen old friendships and form new ones—to dissipate prejudices, to sustain self-respect, and cultivate a spirit of charity and humility, and, through the interchange of opinions and the abrasion of thought, to brush away the cobwebs from the dusty corners of our brains—to sharpen the battle-axe which has grown rusty with disuse, and to lift us out of the monotonous groove of life in which we have been wont to move, bearing pleasant and cheering memories, which, like the Arctic twilight, lingers in the sky to meet the coming dawn of the next returning day.

The present is the third occasion of our meeting in the city of Philadelphia—the earliest medical center of our country, which has long since become the vigorous and lusty sire of many a stalwart son. And here let us congratulate ourselves and the world at large upon the vindication of those principles which have ever guided her medical affairs. As the genuine coin is liable to counterfeit, so have unscrupulous men aimed to speculate upon her fair name by spurious imitations. Thanks to a wise legislation, the atmosphere has been purified, and the parties engaged in the *fraudulent* issue of diplomas have been deprived of their charters, and their institutions abolished.

The first meetings of this Association were marked by harmony and good will throughout; so may it be now, and may the blessing of the "Author of peace and Lover of concord" rest upon us and guide our deliberations.

Once more, then, my friends, offering you our warmest greetings and a hearty welcome to our hospitalities, I give place to your further proceedings.

Dr. Edward Hartshorne, of Pennsylvania, Chairman of the Committee of Arrangements, then said:

*Mr. President and Gentlemen,*—As Chairman of the Committee of Arrangements, I beg leave, in behalf of that Committee, to add our cordial salutation to the eloquent greeting which we have just heard, with so much pleasure, from the Chairman of the Committee of Reception. We are very glad to have reached this stage of the work of preparation, and take great pleasure in reporting the arrangements as sufficiently complete for the purposes of the present meeting.

Not less gratified are we to meet with so large a body of delegates already registered at the opening of the session. It is much the largest number that has thus assembled on any similar occasion. There is every reason for believing, also, that it is as intelligent and well-informed and well-educated a body of our profession as it is a large one.

We can not forget that the sum of knowledge and skill, and superior education, and with these the actual intelligence, are rapidly and greatly increasing in our medical profession, as they are in all the professions and among the people of our country. We must believe, therefore, that this assemblage of the representatives of the medical institutions and medical men from every part and quarter of our country has not come together here for any other purpose and interest than those of science and of our profession, and of a common and truly social brotherhood. Under this impression, we have endeavored to make such arrangements as may satisfy these professional and scientific purposes—such as may encourage those interests and furnish them with food convenient for them.

First, by preparing a museum or collection of objects of scientific and professional character. It is not large or very comprehensive, nor does it pretend to represent the whole and the latest advance even in this city, still less of the country and elsewhere. It is, nevertheless, a collection of which we have no reason to be ashamed. It is one for the excellence of which we are very grateful, and most happy to acknowledge our obligations to contributors and exhibitors, and to the efficient committeemen and aids who have made it the admirable success it has proved itself to be.

Second. With the same or a still greater sense of obligation, we may say still more, if possible, of the exhibition of microscopes and microscopic objects which will be offered to us by the Biological and Microscopical Section of the Academy of Natural Sciences, in the foyer or upper hall, to-night.

During the conduct of the microscopical reception, we will have in the balcony, for the entertainment of those who are in the lower hall, a promenade concert. Then we are to have scientific lectures, with attractive demonstrations, on the succeeding evenings of Wednesday and Thursday. Wednesday lecture by Dr. Noyes, on Ophthalmoscopic Appearances, represented by means of magic lantern transparencies; after this, a lecture by Prof. Rogers, with exhibition of electrical phenommena. Thursday lecture will be given by Dr. J. Solis Cohen, with experiments, on Sound.

On Wednesday evening, the delegates will be received at the houses of Prof. Hodge and Dr. Wm. H. Pancoast. Prof. Joseph Pancoast having been unexpectedly called away from the city under a previous engagement, his son, Dr. Wm. H. Pancoast, has kindly taken his father's place as the host to the Association, and will receive the delegates at his own house, southwest corner of Walnut and Eleventh streets, instead of his father's house, in Chestnut street. On Thursday evening, the delegates will be received by Col. Thomas A. Scott, at his mansion, on the southeast corner of Nineteenth street and Ritten House square. On Friday afternoon, we are to have an excursion to Fairmount Park, with a collation at the Belmont Pavillion. To the excursion and to all of the exhibitions, the ladies with the delegates are most cordially invited. We hope, also, to have them at the exhibition and concert of this evening, and at the museum and the lectures, as well as at the park excursion.

He then presented the following list of delegates and permanent members already registered as in attendance:

There were registered seven hundred and twenty-five delegates, permanent members and members by invitation, from the following States, etc.: Alabama, Arkansas, Colorada, California, Connecticut, Delaware, District of Columbia, Florida, Georgia, Illinois, Indiana, Iowa, Kentucky, Kansas, Maryland, Massachusetts, Minnesota, Missouri, Mississippi, Nebraska, New-Hampshire, New-Jersey, New-York, North-Carolina, Ohio, South-Carolina, Tennessee, Texas, Vermont, Virginia, West-Virginia, Wisconsin, United States Army, United States Navy.

There being no objection to the confirmation of these delegates and permanent members, the President announced that the list thus reported by the Committee on Credentials was approved as read, and the report adopted.

Vice-President Dr. Thos. M. Logan having taken the chair, the President delivered the annual address.

Dr. Henry Askew, of Delaware, offered the following:

*Resolved*, That all questions of a personal character, including complaints and protests, and all questions on credentials, be referred at once, after the report of the Committee of Arrangements, or other presentation, to the Committee on Ethics, and without discussion.

Dr. W. H. Mussey, of Ohio, asked that the motion be divided, referring questions of personal character only to the Committee on Ethics.

Dr. Askew declined dividing the motion.

Dr. Mussey asked if the question did not concern the rights of societies to be admitted in Convention, and he denied the right of the Committee to decide upon such questions. He proposed, also, referring the second branch of the subject to the Convention.

The President said that such a course had never been pursued. Hitherto the Committee on Ethics reported to the Convention on both subjects, their action being open to the approval of the Convention.

Dr. Hartshorne said that the course of the Committee of Arrangements, as a Committee on Credentials, was plain, and defined by law. It was simply to report for confirmation all credentials that could be verified as undoubted and uncontested, and to report for reference all others, together with the protesting or contesting papers filed against them. By admitting delegates and delegations to registration against whom there were objections, the Committee would assume the very right to decide at the expense, and to the probable detriment, of the Association—which is rightly denied by Dr. Mussey, and which belongs, whether affirmatively or negatively, to the Association alone. The Committee had determined to act upon this interpretation of the law, without fear or favor. It was their hope to avoid premature discussion, and thus to secure a fair hearing to all concerned, without any delay beyond what was unavoidable under the action of the meeting and of the Committee on Ethics, to which the questions in dispute were to be referred. The original resolution was framed in accordance with this view. It could not be divided with propriety or justice, because constituencies and representations can not be separated thus; under our organic law, constituencies are established by the admission of their representations, and both may fall together. No question can be more personal than the question of credentials, nor can the supposed rights of the delegates and the constituents, as such, be considered separate, or allowed to regulate each other.

Dr. J. Morris, of Maryland, called the previous question, which being sustained, Dr. Askew's motion was put and carried without a dissenting voice.

Papers were next announced on subjects to be read at the session of the Sections in the afternoon.

The following gentlemen were appointed the Committee on Ethics:

Dr. Henry F. Askew, of Delaware; N. S. Davis, of Illinois;

Calvin Seavy, of Maine; J. K. Bartlett, of Wisconsin; and S. D. Gross, of Philadelphia.

On motion, the Association adjourned until Wednesday at ten A.M.

---

SECOND DAY—WEDNESDAY, May 8, 1872.

Vice-President Dr. T. M. Logan in the chair.

On motion, a recess was taken to permit each State and Territory to select a representative on the Committee on Nominations.

In consequence of the difficulty of hearing the motions, etc., in the hall, the Association reassembled in the First Reformed Presbyterian Church, on Broad street, above Pine.

The President, Dr. Yandell, in the chair.

The Permanent Secretary read the following as constituting the Committee on Nominations:

Alabama, W. O. Baldwin; Arkansas, R. G. Jennings; California, W. R. Cluness; Colorado, W. F. McClelland; Connecticut, S. W. Turner; Delaware, Isaac Jump; District of Columbia, Samuel C. Busey; Georgia, W. F. Westmoreland; Illinois, W. A Knox; Indiana, W. H. Myers; Iowa, W. F. Peck; Kentucky, L. Rogers; Maine, S. Fitch; Maryland, J. Morris; Massachusetts, H. I. Bowditch; Michigan, C. M. Stockwell; Minnesota, Alex. J. Stone; Missouri, Edw. Montgomery; Mississippi, B. A. Vaughan; Nevada, J. C. Tucker; New-Hampshire, J. H. Wheeler; New-Jersey, T. J. Thomason; New-York, Wm. C. Wey; Ohio, C. G. Comegy's; Oregon, Thos. M. Logan; Pennsylvania, R. E. Rogers; Rhode-Island, F. H. Peckham; South-Carolina, R. A. Kinloch; Tennessee, W. Finley; Texas, G. Dowell; Virginia, J. B. McCaw; West-Virginia, R. H. Cummins; Wisconsin, J. T. Reeve; United States Army, J. J. Woodward; United States Navy, G. Clymer.

On motion of Dr. R. E. Rogers, Pennsylvania, the following were elected members by invitation:

Drs. J. Edgar Chancellor, Virginia; M. W. Junkins, Ohio; M. J. Ash, United States Army; W. W. Vinnedge, Indiana; David D. Mabon, Pennsylvania.

The Committee on Nominations were requested to meet at once in Horticultural Hall.

Dr. J. R. Bronson, Massachusetts, offered the following:

*Resolved*, That the Committee on Ethics, to consist of seven members, shall be nominated by the Committee on Nominations.

The President decided that, as an amendment, it must lie over till next year.

Dr. Bronson then offered it as a resolution, to apply to the next meeting. After some discussion, and an appeal from the decision of the Chair, but which decision was sustained by a large vote of the Association, the motion was put and lost by a vote of 167 ayes to 187 nays.

Dr. W. C. Van Bibber, of Maryland, having asked the appointment of a special committee to examine and report upon an unknown remedy which he exhibited, the subject was referred to the Section on Materia Medica and Chemistry.

Dr. N. S. Davis, of Illinois, offered a pamphlet and resolutions touching the action of the Massachusetts Medical Society, which, on motion of Dr. E. L. Howard, of Maryland, were referred to the Committee on Ethics.

Dr. E. Seguin, of New-York, offered some remarks on Clinical Thermometry. On motion, he was requested to bring the subject before the Section on Practice of Medicine and Obstetrics.

The reports of the Committee of Publication and of the Treasurer—the latter showing a balance in the treasury of $1,005—were read and referred to the Committee of Publication.

Dr. J. S. Weatherly, of Alabama, Chairman of the Committee on Medical Education, read his report, which, on motion, was referred to the Committee of Publication.

The Librarian, Dr. F. A. Ashford, of District of Columbia, read his report, which was referred to the Committee of Publication.

In the absence of Dr. Stille, Pennsylvania, Chairman of the Committee on Prize Essays (in consequence of a bereavement), Dr. F. G. Smith, of Pennsylvania, reported that said Committee had awarded but one prize, which was to an essay bearing the motto, "*Netentis aut perfice,*" and entitled, "What Physiological Value has Phosphorus as an Organismal Element?"

On breaking the seal, he announced Dr. Samuel R. Percy, of New-York, as the successful essayist.

On motion, the report and essay were referred to the Committee of Publication.

Dr. Theophilus Parvin, Indiana, Chairman of the Committee of Medical Literature, read his report, which was referred to the Committee of Publication.

Dr. J. D. Jackson, Kentucky, Chairman of the Committee on American Medical Necrology, presented his report, which was, on motion, referred to the Committee of Publication.

The Permanent Secretary announced that Dr. J. G. Richardson, Pennsylvania, would read his report on the Stricture of the White Blood Corpuscles before the Section on Hygiene, Physiology, etc.

A paper by Dr. Henry Hartshorne, Pennsylvania, on the "Physiology of the Vaso-Motor Nerves," was presented and referred to the same Section.

The Secretary also announced that Dr. J. H. Packard, Pennsylvania, would exhibit, at the Section on Surgery and Anatomy, a suspension aparatus for fracture of the leg, and a bracketed splint for excisions of the knee and for compound fractures.

Dr. E. M. Hunt, of New-Jersey, reported progress on the climatology and epidemics of that State.

Dr. George M. Beard, of New-York, offered a paper on "Recent Researches in Electro-Therapeutics, with Demonstrations of Some of the Methods of Application."

On motion, it was referred to the Section on Practice of Medicine and Obstetrics.

Dr. J. P. Garrish, of New-York, offered a paper on "The use of Instruments in Obstetrics," which was similarly referred.

Dr. S. B. Merkel proposed to bring before the Association the case of George Thomas, colored, a native of Brazil, externally well-formed, who moves his heart at will; can also whirl, with an undulating motion, the abdomen like a huge ball around the umbilicus.

On motion, the case was referred to the same Section.

Dr. W. H. Mussey, of Ohio, offered a paper on "Electrolysis in Cancer," and one entitled a "Case of Vesical Calculus and Polypi of the Prostate Gland," illustrated with photographs and specimens.

On motion, both were referred to the Section on Surgery and Anatomy.

A paper on "The Soft Palate in Health and Disease," by Dr. Harrison Allen, of Pennsylvania, and one on "Operation on the Hip-Joint," with a detailed case, by Dr. W. F. Peck, of Iowa, were similarly referred.

Several communications were offered and referred, without comment, to the Committee on Ethics.

Dr. George Sutton, of Indiana, Chairman of the Committee on the Comparative Pathology and the Effects which Diseases of Inferior Animals have upon the Human System, reported progress, and the Committee was continued.

On motion of Dr. A. M. Pollock, of Pennsylvania, it was

*Resolved,* That a Committee of three be appointed by the President to take into consideration the propriety of adopting the suggestions of the Committee on Medical Education.

Dr. J. M. Keller, of Kentucky, offered the following:

*Resolved,* That the Committee on Publication have discretionary power on all papers referred to it, unless instructions to publish accompany the papers when referred.

Dr. J. G. Stetler, of Pennsylvania, offered the following:

*Resolved by the American Medical Association,* That no report, paper, etc., referred by it to the various Sections, shall be referred by the latter to the Committee of Publication, without first having been examined and approved by two-thirds of the members present at said Section.

Dr. F. G. Smith, of Pennsylvania, asked if these were not amendments to the Constitution, and consequently should lie over?

A motion by Dr. P. C. Williams, of Maryland, to lay both on the table, was lost—ayes 104, nays 165.

Dr. S. C. Gordon, of Maine, offered as a substitute:

*Resolved,* That the Committee of Publication have discretionary power as to publishing all papers referred to them by the Society or Sections.

Dr. A. B. Palmer, of Michigan, moved the indefinite postponement of the whole matter.

The President having decided that debate was not in order on such a motion, Dr. J. G. Stetler, of Pennsylvania, appealed from his decision. The question was put and the Chair sustained.

The indefinite postponement was then agreed to by a large majority.

On motion, the Association adjourned to meet on Thursday at ten A.M.

---

THIRD DAY—THURSDAY, May 9, 1872.

The President called the Association to order at ten A.M.

On motion, Drs. E. N. Ash, of North Carolina, and S. Troup Maxwell, of Florida, were elected members by invitation.

The Permanent Secretary read the following communication:

HALL OF THE COLLEGE OF PHYSICIANS OF PHILADELPHIA,
May 1st, 1872.

*W. B. Atkinson, M.D., Permanent Secretary American Medical Association:*

DEAR SIR,—At a stated meeting of the College, held this evening, the following preamble and resolutions were passed:

"WHEREAS, Cases of accidental poisoning and of the internal administration of medicines intended only for external use are as frequent; and whereas, every possible safeguard should be employed to prevent such accidents; therefore,

"*Resolved*, That it is recommended to all druggists to place all external remedies in bottles not only colored, so as to appeal to the eye, but also rough upon one side, so that by the sense of touch no mistake shall be possible even in the dark.

"*Resolved*, That all bottles containing poisons should not only be labeled 'poison,' but also with another label indicating the most efficient and convenient antidote.

"*Resolved*, That a copy of these resolutions be presented to the American Medical Association, the College of Pharmacy of Philadelphia, and the American Pharmaceutical Association, and their assistance asked in bringing about so desirable a reform."

Respectfully, yours, JOHN H. PACKARD,
*Secretary College of Physicians of Philadelphia.*

On motion of Dr. L. A. Sayre, of New-York, these resolutions were unanimously adopted.

On motion of Dr. S. Fitch, of Maine, Dr. Berryman, of England, was invited to a seat with the Association.

Dr. Alex. W. Stein, of New-York, offered the following, which was adopted:

WHEREAS, It has long since been recognized that diseases of a dangerous and fatal nature are transmissible from animals to man, and that certain zymotic affections, which are common to both man and animals, do very frequently manifest themselves first in the latter and subsequently in man—thus warning us that to be indifferent to the condition of the inferior animals is to introduce and create centers of disease among ourselves;

*Resolved,* That a committee be appointed to ascertain what measures can be instituted to prevent the extension of such diseases to man, and what sanitary measures can be effected to arrest the progress of such diseases in animals.

The President appointed as the Committee: Drs. A. W. Stein, New-York; George Sutton, Indiana; and S. D. Gross, Pennsylvania.

Dr. J. M. Keller, of Kentucky, presented a circular, which was at once referred to the Committee on Ethics.

Dr. Frederick Horner, Jr., of Virginia, offered the following, which was adopted:

WHEREAS, The abuse of ardent spirits in our country has proved injurious to the health of the community; therefore,

*Resolved,* That the members of this Association do recommend to our medical brethren to discourage the abuse of alcoholic stimulants in their several communities.

Dr. Francis G. Smith, of Pennsylvania, Chairman of the Committee on Nomenclature of Diseases, reported that, in accordance with instructions given to them by the Association, in 1870, they had prepared a nomenclature to be adopted and observed by the practitioners of the United States. The system presented by the Committee is based upon that of the Royal College of Physicians of London, with such additions and modifications as, in the judgment of the Committee, were needed. In selecting Latin equivalents, the Committee had frequently departed from the English nomenclature, and so far as possible, the English rather than the Latin terms have been employed. The report had appended to it the following:

*Resolved,* That the report of the Committee on Nomenclature of Diseases be referred to a special committee of five members, to be appointed by the President, who shall examine it and report upon its final disposition at the present meeting of the Association.

*Resolved,* That on the favorable report of said Committee, it shall be referred back to the Committee on Nomenclature for the preparation of an index.

Dr. J. J. Woodward, of the United States Army, offered a minority report, as follows:

A minority of the Committee on Nomenclature have the honor to report: That while they entertain the highest respect for the abilities and the learning of those members of the Committee whose residence in Philadelphia has enabled them to attend its meetings and aid in the production of the report which has just been submitted, they nevertheless feel it a duty to express their earnest conviction, that the adoption of a nomenclature and classification by this Association is a matter of too great importance to be acted upon hastily, before the members have had any opportunity to examine for themselves the nomenclature and classification proposed.

The minority of the Committee have had no opportunity to examine the proof-sheets of the work until since the commencement of the present meeting of the Association, and the time since then has been far too short for them to form an opinion as to details. They do not, therefore, wish to be understood as criticising the work done in any way, but simply ask, as a measure of justice and wisdom, that it may be submitted to the judgment of the profession before it is acted upon by this body.

We have the honor, therefore, to offer the following resolution, as a substitute for the resolution offered by the majority:

*Resolved,* That the nomenclature and classification just submitted by the Committee be published in the Transactions; that one thousand extra copies be printed in pamphlet form and distributed to the profession, and that the question of the adoption of the nomenclature and classification by this body be postponed till the next annual meeting.

On motion of Dr. A. B. Palmer, of Michigan, both reports were accepted.

The reports were discussed by Drs. Woodward, Hamilton, White, Howard, Gallaher, Newman, Palmer, Atlee and Squibb.

Dr. Woodward's resolution was then adopted.

Dr. J. M. Keller, of Kentucky, offered the following, which was not adopted:

*Resolved,* That so much of the report of the Committee on Medical Education as referred to the establishing of a Medical Congress, consisting of an equal number of representatives from each State, is of immediate importance; and in order to a furtherance of the views expressed, that a committee of one from each State be appointed at once, to take the preliminary steps necessary to organizations, with instructions to report before adjournment of the present session.

Dr. Baldwin read a partial report from the Committee on Nominations, as follows:

*President*—Dr. Thomas M. Logan, California.
*First Vice-President*—Dr. B. H. Catlin, Connecticut.
*Second Vice-President*—Dr. W. M. McPheeters, Missouri.
*Third Vice-President*—Dr. A. M. Pollock, Pennsylvania.
*Fourth Vice-President*—Dr. W. T. Biggers, Tennessee.
*Treasurer*—Dr. Caspar Wister, Pennsylvania.
*Librarian*—Dr. William Lee, District of Columbia.

*Committee on Library*—Dr. J. M. Toner, District of Columbia.
*Assistant Secretary*—Dr. M. A. Pallon, Missouri.
Next place of meeting—St. Louis.

Dr. Baldwin requested further time for his Committee to complete their report.

On motion, the report was adopted.

Dr. M. A. Pallon, of Missouri, thanked the Association for having thus handsomely agreed to meet next year in St. Louis. He presented, in behalf of Dr. S. Gratz Moses, of St. Louis, who was absent, the following plan to elevate the standard of medical men:

WHEREAS, The vast extent and increasing population of this great country creates an increased demand for medical men, numerous medical schools have sprung up in every direction, nearly all of which have the power of granting diplomas to their graduates, and that a diploma is simply a certificate that its recipient has passed an examination satisfactory to the faculty of said schools; therefore, it behooves the medical profession to grant a higher reward of merit by such of its members who may be distinguished by high medical attainments and great moral worth; therefore, be it resolved—

1. That the American Medical Association of the United States take means to establish a National Academy of Medicine.

2. That the object of the Academy of Medicine shall be to elevate the standard of medical attainments and moral worth of the profession, so that a certificate of membership of the Academy shall be the highest evidence and mark of distinction of a learned and honorable physician.

3. To accomplish these objects, that a Board or Boards of Examiners, without salary, and irrespective of medical schools, shall be yearly appointed by the National Academy, whose duty it shall be to examine carefully and conscientiously each applicant for membership.

4. That the Board or Boards shall report those who have passed a satisfactory examination to the National Academy of Medicine, at their yearly sessions, who shall proceed to ballot for the candidates.

5. To all those who shall be elected, a certificate of membership shall be granted, which certificate shall be signed by the President and Secretary of the Academy of Medicine.

6. That the cost of said certificate shall be ———.

7. That the amount accruing from the above source shall compose a fund to be properly invested and held in trust for the benefit of the needy widows and orphans of deceased members of the National Academy of Medicine.

Dr. Pallon then moved that a committee of seven be appointed by the President to carry out the object of these resolutions, and that their author, Dr. S. Gratz Moses, of St. Louis, be Chairman.

This motion was lost, and no further action was taken on the subject.

Dr. S. Troup Maxwell, of Florida, presented a laryngoscope, which was referred to the Section on Surgery and Anatomy.

Dr. T. M. Logan, of California, Chairman of the Committee on National Health Council, presented a report, and asked to be continued, to constitute a special Section on State Medicine.

On motion, the report was received.

Dr. J. C. Tucker, California, offered as a substitute a resolution asking Congress to establish a National Sanitary Bureau, and presented an act bearing on this subject.

On motion of Dr. T. J. Gallaher, of Pennsylvania, this resolution was laid on the table.

Dr. R. A. Kinloch, of South Carolina, offered an amendment to the resolution attached to Dr. Logan's report, striking out the appeal to the Government, which was accepted by Dr. Logan, and the Committee was continued in accordance with the report of the Chairman.

Dr. T. J. Gallaher, of Pennsylvania, offered the following amendment to the Constitution:

*Resolved*, That the United States Marine Hospital service be placed in the same relative position in the Association as the United States Army and Navy Hospital service, and that the Constitution and By-Laws of the Association be amended as follows:

In paragraph two of the second section of the Constitution, after the words "Army and Navy," insert, "and the United States Marine Hospital service."

In the sixth section of the By-Laws, after the words "the Chiefs of the Bureaus of the Army and Navy," insert, and "the Supervising Surgeon of the United States Marine Hospital service."

Under the rules, this lies over till next year.

Committee on Nominations presented their additional report:

*Committee of Arrangements.*—Drs. J. B. Johnston, J. T. Hodgen, John S. Moore, Robinson, Kennard, Leete, Brokaw, J. M. Scott—all of St. Louis.

*Commitee of Publication.*—Drs. F. G. Smith, Chairman, Pennsylvania; W. B. Atkinson, Pennsylvania; D. Murray Cheston, Pennsylvania; William Lee, District of Columbia; Caspar Wister, Pennsylvania; H. F. Askew, Delaware; J. Aitken Meigs, Pennsylvania.

*Committee on Prize Essays.*—Drs. John S. Moore, Chairman, St. Louis; E. S. Gregory, St. Louis; N. S. Davis, Chicago; Theophilus Parvin, Indiana; George Mendenhall, Cincinnati.

*Committee on Medical Education.*—Drs. Wm. Carson, Chairman, Cincinnati, Ohio; Joseph P. Logan, Georgia; E. Lloyd Howard, Maryland; H. K. Steele, Colorado; S. Oakley Vanderpoel, New-York.

*Committee on Medical Literature.*—Drs. Austin Flint, Chairman, New-York; L. P. Yandell, Sr., Kentucky; Henderson, Alabama; S. B. Thrall, Iowa; C. Seavy, Maine.

*Committee on Medical Necrology.*—Drs. J. D. Jackson, Chairman, Kentucky; Charles W. Parsons, Rhode-Island; E. A. Hildreth, West-Virginia; W. W. Johnston, District of Columbia; G. L. Simmons, California; W. C. Warriner, Oregon; E. B. Stevens, Ohio; D. Hayes Agnew, Pennsylvania; D. W. Stormont, Kansas; J. B. Johnson, Missouri; H. R. Storer, Massachusetts; W. S. W. Ruschenberger, United States Navy; E. H. Hazen, Iowa; Levi G. Hill, New-Hamshire; A. Sager, Michigan; V. Kersey, Indiana; A. E. Ames, Minnesota; W. H. Newman, Colorado; J. O. Hamilton, Illinois; J. H. Peabody, Nebraska; L. P. Bush, Delaware; G. W. Russell, Connecticut; Samuel C. Chew, Maryland; S. H. Stout, Georgia; T. J. Heard, Texas; A. H. Scott, Arkansas; William H. Bailey, New-York; B. R. Jones, Alabama; D. McRuer, Maine; A. N. Talley, South-Carolina; John D. Blaine, New-Jersey; J. W. M. Shattuck, Missouri; Levin S. Jones, Virginia.

OFFICERS OF SECTIONS.

*Chemistry and Materia Medica.*—Drs. R. E. Rogers, Philadelphia, Pennsylvania, Chairman; Ephraim Cutter, Boston, Massachusetts, Secretary.

*Practice of Medicine and Obstetrics.*—Drs. D. A. O'Donnell, Baltimore, Maryland, Chairman; Benj. F. Dawson, New-York, Secretary.

*Surgery and Anatomy* —Drs. Edw. Warren, Baltimore, Chairman; W. F. Peck, Davenport, Iowa, Secretary.

*Meteorology and Epidemics.*—Drs. George Sutton, Aurora, Indiana, Chairman; Elisha Harris, New-York, Secretary.

*Medical Jurisprudence, Hygiene and Physiology.*—Drs. S. C. Busey, Washington, Chairman; A. B. Arnold, Baltimore, Secretary.

*Psychology.*—Drs. Isaac Ray, Chairman, Philadelphia; John Curwen, Secretary, Harrisburg.

Several resolutions relative to the publication of papers in the Transactions and instructing the Committee of Publication were offered; but after discussion, on motion of Dr. N. S. Davis, of Illinois, the whole matter was indefinitely postponed.

The Committee on Ethics reported, through Dr. N. S. Davis, as follows:

Your Committee have made a careful and patient examination of each and every subject referred to them by the Association. In all cases involving the rights of individuals or organized institutions to representation, the individuals and the representatives of such institutions have been invited before the Committee, and their testimony carefully taken. As a result of these investigations, the Committee ask leave to submit the following unanimous report:

First. In relation to the preamble and resolutions offered by Dr. Davis, touching the Massachusetts Medical Society, your Committee recommend them for unanimous adoption by the Association. They are as follows:

WHEREAS, It has been represented that the Massachusetts Medical Society considers that its delegations to the annual meeting of the American Medical Association in Washington, May, 1870, were unjustly excluded by the Committee of Arrangements;

AND WHEREAS, The action of the Committee on Ethics, at the same meeting, in refusing to allow the right of said Committee of Arrangements to exclude the Massachusetts delegation, is not yet fully understood by that Society; therefore,

*Resolved*, That this Association acknowledge the great and effective efforts of the Massachusetts Medical Society to elevate the profession, and to suppress quackery of all sorts, and especially assure that Society of encouragement and support in its present exertions to rid itself of all pretenders.

*Resolved*, That the said Association trusts that hereafter there will be a united and harmonious action of all the several bodies of which the Association may be composed.

*Resolved*, That a copy of these resolutions be forwarded to the President of the Massachusetts Medical Society.

This was agreed to by the Association.

The Committee report in regard to the official communication of the Corresponding Secretary of the Medical Association of the District of Columbia, certifying that Drs. J. D. Barnes, S. S. Bond, A. McWilliams, W. E. Poulton, S. B. Blanchard, S. W. Caldwell, J. L. Crouse, James Phillips and G. Sylvester have forfeited their membership in that Society by reason of not having paid their dues for three years, and after repeated notices of the fact and its consequences; your Committee recommend that their names be stricken from the roll of membership, in accordance with the fifth paragraph of the second section of the Constitution of this Association; and also the same action in regard to Dr. D. W. Bliss, who is under sentence of expulsion from that Society.

Unanimously adopted.

In regard to alumni associations of medical colleges, the Committee reported that it did not consider them such medical societies as were intended by the Constitution to be eligible to membership; and hence they recommend that no delegates be received from any of the alumni associations of any of the medical colleges from any part of the country.

Unanimously agreed to.

In regard to the Pathological Society of Berks county, Pennsylvania, the registration of whose delegates had been postponed on account of the protest alleging the want of good standing on the part of that Society, the Committee postponed action from the want of proper evidence.

On motion, this request was granted, and further action on this institution was postponed.

In regard to the Academy of Medicine of Washington, D. C., the Freedman's Hospital of the District of Columbia, and the Howard University of Washington, D. C., the registration of whose delegates had been postponed by the Committee of Arrangements, on account of want of good standing on the part of these institutions, as indicated by the action of this institution in 1870 and 1871, and by information communicated to that Committee, we report the facts as follows:

1. That this Association, at its meeting in San Francisco, in 1871, by the emphatic vote of eighty-three to twenty-six, refused to so amend the Constitution as to admit delegates from colleges in which women are taught and graduated in medicine, and from hospitals in which women, graduates in medicine, attend.

2. That this Association, in 1870, declared, by an almost equally emphatic vote, that a medical society, constituted in part of members who are not licensed to practice in accordance with the civil law governing the practice of medicine in the State or district in which the society is located, is not entitled to representation in this Association.

3. That sections 3, 4 and 5 of the Act of Congress, passed July, 1868, incorporating the Medical Society of the District of Columbia, and which has been the law regulating the practice of medicine in that District up to the present time, requires all persons, coming into the District to practice medicine, to apply for, and within six months obtain, a license to practice, from a Board of Examiners appointed for that purpose, and makes it a misdemeanor, punishable by a fine of $50, for every act of practice without such license.

4. That it has been proven, by the testimony of several witnesses, that the Medical Society of the Academy of Medicine of Washington now contains, in full fellowship, at least four or five members who have never applied for and obtained license to practice, and yet are actually practicing medicine, and three of whom are on the list of delegates sent by that Society to this Association; also, that one of them is a member of the medical staff of the Freedman's Hospital, and also that several of the faculty of the Howard University are members of the same Academy of Medicine, and one of the teachers is a woman.

In view of these facts, the Committee can not regard either of the three institutions named as in good standing, whether tested by civil law, by the former decisions of this Association, or by its Code of Ethics; and hence, the Committee recommend that the delegates from those several institutions be not received into this body.

Dr. Reyburn, of Washington, D. C., obtained the floor, but granted Dr. Davis permission to read the next and final section of the report, which was in reference to the resolution of Dr. Finley, asking of this Committee a clear interpretation of what is now a part of the by-laws of the Association.

The Committee offered the following:

*Resolved,* That members of the profession hired by the month or year for definite wages, by families, railroads, manufacturing incorporations, or any money-making institution whatever, for ordinary surgical or medical practice—always excepting eleemosynary and charitable institutions and hospitals—are to be classed as irregular practitioners, and, therefore, disqualified for membership in this Association, or in State or county societies.

Dr. Weatherly moved to refer this question back to the State societies. Agreed to.

Dr. R. Reyburn, of District of Columbia, spoke in defense of himself and the Howard University, etc.

Dr. S. C. Busey, of District of Columbia, replied in defense of the Licensing Board at Washington, etc.

Dr. J. R. Bronson, of Massachusetts, asked if colored physicians were licensed by this Board.

Dr. Busey replied that they were.

Dr. G. S. Palmer, of District of Columbia, spoke in defense of himself, the Howard University, etc.

Dr. Edward Hartshorne, of Pennsylvania, replied to Dr. Reyburn, and proved incontestably that he had been warned before leaving Washington.

The previous question was now demanded and sustained.

On motion of Dr. L. A. Sayre, of New-York, the report of the Committee on Ethics was received and adopted by a very large majority.

On motion, the Association adjourned until Friday, May 10, at ten A.M.

---

Fourth Day—Friday, May 10, 1872.

The Association was called to order at ten A.M., by the President.

Dr. A. B. Palmer, of Michigan, offered the following, which was unanimously adopted:

Whereas, It has pleased the Great Ruler of the Universe to take from us our former President, Dr. Zina Pitcher, of Detroit—a man of large attainments, of unspotted character, of great benevolence, and unusual devotion to the profession of his choice; therefore,

*Resolved,* That in the death of Dr. Pitcher the profession has lost one of its purest members, the community one of its greatest benefactors, and this Association one of its most dignified and genial members.

*Resolved,* That we mingle our sympathy with the family and the immediate friends of the deceased, and that a copy of these resolutions be transmitted by the President of the Association to his bereaved family.

Dr. P. Pineo, of Massachusetts, offered the following amendment to the Constitution, which was laid over till next year:

*Resolved,* That the United States Marine Hospital service be placed in the same relative position in the American Medical Association as the Medical Department of the United States Army and Navy; and that in paragraph two of the second section of the Constitution, after the words "Army and Navy," the words, "and the United States Marine Hospital Service," be inserted. Also, in section six of the by-laws, after the words, "the Chief of the Bureaus and of the Army and Navy," be inserted, "and the Supervising Surgeon of the United States Marine Hospital Service."

The Committee on Nominations presented their final report, which was, on motion, adopted:

SPECIAL COMMITTEE.

*Committee on Vaccination.*—Dr. T. N. Wise, of Kentucky.

*Committee on Diseases of Colorado.*—Dr. John Elsner, of Denver, Colorado.

*Committee on the Treatment of Fractures.*—Dr. Lewis A. Sayre, of New-York.

*Committee on Chua Bibberina, a Substitute for Quinia.*—Dr. Wm. Chew Van Bibber, of Maryland.

*Committee on Gynæcology.*—Dr. Montrose A. Pallen, of Missouri.

*On the Climatology and Epidemics*—Of New-Hampshire, Dr. G. R. Crosby; Vermont, G. E. Bullard; Massachusetts, Ephraim Cutter; Rhode-Island, Ed. T. Caswell; Connecticut, J. C. Jackson; New-York, Gouverneur M. Smith; New-Jersey, E. M. Hunt; Pennsylvania, W. L. Wells; Maryland, C. H. Ohr; Georgia, Geo. M. McDowell; Missouri, T. B. Lester; Alabama, T. C. Osborn; Texas, S. M. Welsh; Illinois, David Prince; Indiana, Dugan Clark; District of Columbia, J. W. H. Lovejoy; Iowa, G. M. Staples; Michigan, S. H. Douglas; Ohio, J. A. Murphy; California, W. H. Williams; Tennessee, W. K. Bowling; West-Virginia, H. W. Brock; Minnesota, Charles H. Hewitt; Virginia, —— Claiborne; Delaware, L. P. Bush; Kansas, Tiffin Sinks; Mississippi, S. V. D. Hill; Louisiana, S. M. Bemiss; Wisconsin, J. K. Bartlett; Kentucky, L. P. Yandell, Sr.; Colorado, R. G. Buckingham; Oregon, Horace Carpenter; North-Carolina, E. Burke Haywood; South-Carolina, M. Simmons; Maine, J. H. Tewksbury.

The President appointed the following Committee on Publication of a National Medical Journal, as previously agreed to:

Drs. A. M. Pollock, Pennsylvania; W. F. Westmoreland, Georgia; A. N. Talley, South-Carolina; J. Walker, New-York; J. D. Jackson, Kentucky; J. S. Weatherly, Alabama; and H. Maguire, Virginia.

Dr. J. G. Stetler, of Pennsylvania, offered the following, which, on motion, was laid on the table:

WHEREAS, The American Medical Association did refer the whole subject of contract physicians—after the report of the Committee on Ethics on the subject—to the various State medical Societies; therefore,

*Resolved,* That everything relating to this subject be rescinded or stricken from the ordinances of this Association.

The report of Dr. Hewitt, on the Climatology of Minnesota, presented too late for action by the Section on that subject, was referred to the Committee of Publication.

A voluminous paper on Yellow Fever, by Dr. Jos. Jones, of Louisiana, was presented from the Section on Practice of Medicine and obstetrics, with the report that it was impossible for said Section to properly examine it in the short time at their disposal.

On motion of Dr. C. Wister, of Pennsylvania, it was

*Resolved,* That the paper of Dr. Jones, of New-Orleans, be returned to the author, with the request that he will abridge his paper, if possible; and if not, present it next year to the Chairman of the Section on Practice, a day previous to the meeting, as directed by law, so that it may have due consideration.

The Section on Chemistry and Materia Medica reported their minutes, which, on motion, with the accompanying papers, was referred to the Committee of Publication.

Two papers offered by Dr. C. F. Perkins, of Pennsylvania, were referred back to their author, as the Section had not time to properly examine them.

Dr. L. P. Bush, of Delaware, offered the following, which, on motion of Dr. Porter, of Delaware, was adopted by a standing vote:

The removal, in the order of Divine Providence, by death, of Dr. W. W. Gerhard, of this city, marks an event of more than ordinary importance; therefore,

*Resolved,* That the combination of high talents with education in careful and extensive observation, and close analysis, as preliminary to his entering upon the practice of medicine, pursued with extraordinary diligence under the most favorable advantages, in the Hotel Dieu, in the clinic of that great master, Baron Louis, perfected him in the diagnosis especially of the diseases of the heart and lungs, and of typhoid fever; and added to this, his pathological knowledge, brought with him from abroad, was so remarkably accurate, and so admirably and successfully were these accomplishments exhibited to his classes in the Biockley Hospital, the first field of his labors after his return home, and afterward in the Pennsylvania Hospital, that they could not fail to imbue the classes with the enthusiasm which manifested itself in his teachings by the autopsy. The profession of that day, without hesitation, accorded to him, though a young man, a position *primus inter pares*, and hence his reputation rapidly spread through the country, and brought to him from all sections those who desired the benefit of his wise judgment in disease.

*Resolved*, That this Association desires to record thus its high appreciation of the services and character of this lamented member of the profession, and that a copy of these resolutions be transmitted to the family of the deceased.

Dr. L. J. Deal, Chairman of the Committee on Cultivation of the Cinchona Tree in the United States, presented a report, which was adopted and referred to the Committee of Publication.

The special committees were called as follows:

On the Anatomy and Diseases of the Retina, Dr. R. F. Michel, Alabama, Chairman. No report.

On the Comparative Pathology and the Effects which Diseases of Inferior Animals have upon the Human System, Dr. George Sutton, Indiana, Chairman, reported progress, and was continued.

On the Structure of White Blood Corpuscles, Dr. J. G. Richardson, Pennsylvania, Chairman, had already read his report to a Section, by whom it was referred to the Committee of Publication.

On Vaccination, Dr. T. N. Wise, Kentucky, Chairman. No report.

On Skin Transplantation, Dr. J. Ford Thompson, District of Columbia, Chairman, was, on motion, continued.

On the Nature and Process of the Restoration of Bone, Dr. A. L. McArthur, Illinois, Chairman. No report.

On Some Diseases Peculiar to Colorado, Dr. John Elsner, Colorado, Chairman, had already been continued by the Committee on Nominations.

On Correspondence with State Medical Societies, Dr. N. S. Davis, Illinois, Chairman, reported as follows:

Your Committee, in compliance with the instructions of the Association at the last annual meeting, have sent printed copies of their report last year to the Secretaries of the several State medical societies, asking them to call the attention of their respective societies specially to the resolution appended to the report relating to the subject of the preliminary education of those proposing to study medicine.

Such of the State societies as have held their meetings since the communication was sent, have given indications of most gratifying progress in the development of a proper professional sentiment on that subject. But as many of the societies have not had time to report, we would simply report progress, and ask to be continued.

On What, if any, Legislative Means are Expedient and Advisable to Prevent the Spread of Contagious Diseases, Dr. M. H. Henry, New-York, Chairman. No report.

On the Climatology and Epidemics—Of Massachusetts, Dr. E. Cutter; New-York, Dr. W. F. Thoms; New-Jersey, Dr. E. M. Hunt; Pennsylvania, Dr. W. L. Wells; Missouri, Dr. W. S. Edgar; Texas, Dr. S. M. Welsh; California, Dr. F. W. Hatch; West-Virginia, Dr. E. A. Hildreth; Minnesota, Dr. Chas. N. Hewitt; Delaware, Dr. L. P. Bush; Wisconsin, Dr. J. K. Bartlett; Colorado, Dr. R. G. Buckingham; Oregon, Dr. E. R. Fiske; South-Carolina, Dr. M. Simmons. Reports, either in full or of progress, were presented and referred to the Section on Climatology and Epidemics during the session.

On motion of Dr. J. S. Weatherly, Alabama, it was

*Resolved,* That the Committee appointed to consider the report on Medical Education be allowed to report at our next meeting.

Dr. S. D. Gross, Pennsylvania, offered the folllowing:

SECTION III.—STANDING COMMITTEES.

*Amendment to the By-Laws.*

That instead of a report on Medical Education, on Medical Literature, Climatology and Epidemic Diseases, there shall annually be delivered before the Association, at its general meetings, an address in Medicine, in Surgery, and an address in Midwifery, or the Diseases of Children: the lecturers to be appointed this year by the President—afterward by the Committee on Nominations.

After some discussion, on motion of Dr. A. B. Palmer, Michigan, it was laid over for consideration at the next session.

On motion of Dr. E. L. Howard, Maryland, it was

*Resolved,* That a committee of three be appointed to report, at the next meeting of the Association, a plan for the better arrangement of the Sections, and for the more rigid examination of papers offered for publication.

Committee: Drs. E. L. Howard, Maryland; J. R. Bronson, Massachusetts; R. E. Rogers, Pennsylvania.

The following preamble and resolutions, offered by Dr. Henry Hartshorne, Pennsylvania, at the Section on Chemistry and Materia Medica, and by them adopted, were, on motion, unanimously adopted by the Association:

Whereas, In all capital criminal trials involving questions of medical jurisprudence, there is an obvious disadvantage in the testimony of the scientific experts being made to appear partial or antagonistic, by their being employed as witnesses upon one or the other side; therefore,

*Resolved,* That it is the sense of this Association that, in important criminal cases requiring the evidence of medical or chemical experts, the cause of justice will be promoted by the appointment by the court, in every such case, of a commission of experts empowered to collect all purely scientific testimony bearing on the case, and report upon it to the court by which the case is to be tried.

*Resolved,* That by the appointment of such scientific commission, the present system of summoning chemical and medical witnesses in criminal trials might be dispensed with to advantage.

*Resolved,* That the same recommendation applies also to cases of surgical or medical malpractice.

*Resolved,* That the State Associations be requested to bring this matter, at an early date, before their respective Legislatures.

The Section on Meteorology, Medical Topography and Epidemics, reported their minutes, which, with the accompanying papers, were, on motion, referred to the Committee of Publication.

On motion of Dr. D. M. Cheston, of Pennsylvania, it was

*Resolved,* That Dr. H. F. Askew, of Delaware, be requested to prepare suitable resolutions relative to the late Prof. Samuel Jackson, M.D., for entry on the minutes.

Dr. W. F. Peck, of Iowa, offered the following amendment to the Constitution, which laid over under the rules:

Amend section two of the Constitution so as to contain the words "Marine Hospital Service" after the words "Army and Navy."

Dr. C. Percy LaRoche, of Pennsylvania, offered the following, which, on motion, was laid on the table:

*Resolved,* That the American Medical Association invite their medical brethren throughout the civilized world to join them in a Medical Congress, to be held in 1876, at such place designated by the Association at its next meeting in St. Louis.

On motion of Dr. Alex. W. Stein, of New-York, it was

*Resolved,* That the Committee appointed at the meeting of this Association held at Washington, May, 1870, to report on what means are expedient to prevent the renewal of prescriptions by druggists without the consent of the physician ordering said prescription, be continued another year.

On motion of Dr. Edward Hartshorne, Pennsylvania, it was

*Resolved,* That the thanks of this Association are presented to the officers and proprietors of the various public institutions and other establishments, and to the officers in command of the United States Navy-Yard, and the League Island Station, in Philadelphia, in acknowledgment of the kind invitations extended to the Association.

*Resolved,* That the cordial thanks of this Association are due, and are hereby presented, to the authorities and congregation of the First Reformed Presbyterian Church, in Philadelphia, for their timely and generous permission to occupy their building.

On motion of Dr. Edward Hartshorne, Pennsylvania, it was

*Resolved,* That the honor which has been accorded to the memory of Dr· W. W. Gerhard is eminently due, and is hereby offered, to those of the late Prof. S. Henry Dickson, of Jefferson Medical College, and to the late Prof. Samuel Jackson, of the University of Pennsylvania, both of whom have recently deceased. Their names and reputation, like that of Dr. Gerhard, are identified with the history of medicine in America. The American Medical Association owes them a record of grateful recognition, which would be as much an honor to itself as to its memory.

*Resolved,* That copies of this resolution be sent to the families of the deceased.

On motion of Dr. Edward Hartshorne, Pennsylvania, it was

*Resolved,* That Dr. J. M. DaCosta, Pennsylvania, be appointed a committee to prepare an appropriate notice of the death of Prof. Dickson, to be placed upon the minutes and to be conveyed to the faculty.

Dr. Henry M. Skillman, Kentucky, offered the following:

WHEREAS, Dr. W. B. Atkinson has served the Association for eight years, in the arduous office of Permanent Secretary, without any sufficient remuneration;

AND WHEREAS, His duties must become more engrossing every year, and their proper performance must interfere with other more profitable employment, and entail labor upon him well deserving compensation; be it

*Resolved,* That he receive the thanks of the Association for his past services, and that the occupant of the office of Permanent Secretary shall hereafter be paid a salary of one thousand dollars per annum, in quarterly payments.

After some discussion, on motion of Dr. N. S. Davis, this matter was referred to a special committee of three, to be appointed by the President, to report at the next meeting.

Committee: Drs. N. S. Davis, Illinois; J. P. White, New-York; Edward Hartshorne, Pennsylvania:

On motion of Dr. Edward C. Harwood, of New-York, it was unanimously

*Resolved,* That we return our grateful thanks to our Permanent Secretary for his valuable services; and as a more permanent testimonial to his merit, we present him the sum of $500.

On motion of Dr. T. M. Logan, of California, it was

*Resolved,* That the thanks of the Association be extended to the Committee of Arrangements, the members of the medical profession of Philadelphia, the Professors of the Jefferson Medical College and of the University of Pennsylvania, and to the citizens generally, and especially to Col. Thomas A. Scott, for the kind attention and hospitalities extended to the members of this Association.

The following, offered by Dr. Henry Hartshorne, of Pennsylvania, was laid on the table:

*Resolved,* That reaffirming the resolution of 1870, by this Association, recognizing Horace Wells as the discoverer of the practical application of anæsthesia for surgical purposes, we hereby acknowledge the good will and sense of justice evinced by Sir James Y. Simpson and others in England toward an American discoverer, which led to originating in England the "Horace Wells Testimonial Fund," and we cordially recommend a movement in this country for the same purpose.

On motion of Dr. L. S. Bolles, of Pennsylvania, it was

*Resolved,* That the resolution offered by Dr. Askew, of Delaware, and adopted by the Association—to wit, "that all questions of a personal character, including complaints and protests, and all questions of credentials, be referred at once to the Committee on Ethics, and without discussion"—be adopted by the Association as a standing resolution.

The minutes of the Section on Surgery were received, and, with the accompanying papers, were, on motion, referred to the Committee of Publication.

Dr. Jas. Parsons, of New-York, offered the following, which was adopted:

Whereas, Paluel de Marmon has been permitted to register as a delegate from the Westchester County Meuical Society of New-York;

And Whereas, He was appointed nearly ago, and while he was supposed to be in good standing, but since that time his name has been stricken from the Yonkers' Medical Association; he has been refused admission to the New-York Academy of Medicine, and charges will be brought against him, for unprofessional conduct, at the annual meeting of the Westchester County Medical Society; therefore,

*Resolved,* That the name of Paluel de Marmon be expunged from the register, and the case referred to the Committee on Medical Ethics, with instructions to report at the next annual meeting of this Association.

On motion of Dr. W. O. Baldwin, of Alabama, it was

*Resolved,* That a special Committee on the Relations Between Physicians and Druggists be established, and that Dr. R. J. O'Sullivan be appointed Chairman of said Committee, with a request to report at the next meeting of the Association.

Dr. J. M. Toner, District of Columbia, offered the following, which was laid on the table:

*Resolved,* That a committee of three be appointed by the Chair to consider and report at the next meeting of the Association a suitable design for a medal of membership.

Dr. W. S. Huselton, Pennsylvania, offered the following, which, on being put by the Vice-President, Dr. T. M. Logan, was unanimously adopted:

*Resolved,* That the thanks of the Association are tendered the retiring President, Dr. Yandell, for the able and impartial manner in which he has presided over our deliberations during the present session.

The President appointed as delegates to the British Medical Association, Drs. Charles A. Hart, New-York; John Davies Jackson, Kentucky; J. R. Bronson, Massachusetts; F. Henderson; J. F. Johnston, Alabama; J. M. DaCosta, Pennsylvania.

Dr. W. W. Reese, New-York, offered the following, which was laid on the table:

*Resolved,* That whilst we admit the right of women to acquire medical education, and to practice medicine and surgery in all their departments, we deem the public association of the sexes in our medical schools and at the mixed clinics of our hospitals as impracticable, unnecessary, and derogatory to the instincts of true modesty in either sex.

On motion of Dr. T. J. Gallaher, Pennsylvania, it was

*Resolved,* That the thanks of this Association be tendered to Drs. W. H. Pancoast and Hugh L. Hodge, for their very elegant and sumptuous entertainments given to the members of this body.

On motion of Dr. R. W. Gibbes, South-Carolina, it was

*Resolved,* That the thanks of the American Medical Association be tendered to those railroad companies which have kindly reduced their rates of fare to our delegates.

The following, offered by Dr. I. N. Quinby, of New-Jersey, was laid on the table:

Whereas, Fatal results do sometimes occur from physicians neglecting to write on their prescriptions how the medicines are to be taken; therefore,

*Resolved,* That the safety of the community requires that in all cases in which active remedies are prescribed for external use, the apothecary to whom the prescription is sent be requested by the prescriber to write legibly on the phial, or other receptacle containing the medicine prescribed, the precise dose or manner of using the same.

On motion of Dr. R. E. Rogers, Pennsylvania, the resolution of Dr. Reese, relative to the teaching of female students at the same hours and place with males, was taken from the table and again considered. After some discussion, on motion of Dr. Edward Hartshorne, it was indefinitely postponed.

On motion of Dr. J. S. Morris, of Maryland, it was

*Resolved,* That the thanks of the Association be tendered to the members of the Press of Philadelphia for their faithful reports of our proceedings.

Dr. N. S. Davis then moved, that after the President had given a farewell address, the Association should adjourn to meet in St. Louis, on the first Tuesday in May, 1873.

The President then thanked the members for their kindness and courtesy to him, and declared the Association adjourned until 1873.

